# Emergence of antibiotic-specific *Mycobacterium tuberculosis* phenotypes during prolonged treatment of mice

**DOI:** 10.1128/aac.01310-24

**Published:** 2025-01-17

**Authors:** Elizabeth A. Wynn, Christian Dide-Agossou, Reem Al Mubarak, Karen Rossmassler, Victoria Ektnitphong, Allison A. Bauman, Lisa M. Massoudi, Martin I. Voskuil, Gregory T. Robertson, Camille M. Moore, Nicholas D. Walter

**Affiliations:** 1 Rocky Mountain Regional VA Medical Center 19982 , Aurora, Colorado, USA; 2 Center for Genes, Environment and Health, National Jewish Health 2930 , Denver, Colorado, USA; 3 Consortium for Applied Microbial Metrics, Aurora, Colorado, USA; 4 Division of Pulmonary Sciences and Critical Care Medicine, University of Colorado Anschutz Medical Campus 199566 , Aurora, Colorado, USA; 5 Linda Crnic Institute for Down Syndrome, University of Colorado Anschutz Medical Campus 637889 , Aurora, Colorado, USA; 6 Mycobacteria Research Laboratories, Department of Microbiology, Immunology, and Pathology, Colorado State University 164598 , Fort Collins, Colorado, USA; 7 Department of Immunology and Microbiology, University of Colorado Anschutz Medical Campus 549224 , Aurora, Colorado, USA; 8 Department of Biostatistics and Informatics, University of Colorado Anschutz Medical Campus 129263 , Aurora, Colorado, USA; St. George's, University of London, London, United Kingdom

**Keywords:** antibiotic exposure, *M. tuberculosis*, RNA-sequencing, antibiotic resistance

## Abstract

A major challenge in tuberculosis (TB) therapeutics is that antibiotic exposure leads to changes in the physiology of *M. tuberculosis* (*Mtb*), which may enable the pathogen to withstand treatment. While antibiotic-treated *Mtb* has been evaluated in *in vitro* experiments*,* it is unclear if and how long-term *in vivo* treatment with diverse antibiotics with varying treatment-shortening activity (sterilizing activity) affects *Mtb* physiologic processes differently. Here, we used SEARCH-TB, a pathogen-targeted RNA-sequencing platform, to characterize the *Mtb* transcriptome in the BALB/c high-dose aerosol infection mouse model following 4 weeks of treatment with three sterilizing and three non-sterilizing antibiotics. Certain transcriptional changes were shared among most antibiotics, including decreased expression of genes associated with protein synthesis and metabolism and the induction of certain genes associated with stress responses. However, the magnitude of this shared response differed between antibiotics. Sterilizing antibiotics rifampin, pyrazinamide, and bedaquiline generated a more quiescent *Mtb* state than did non-sterilizing antibiotics isoniazid, ethambutol, and streptomycin, as indicated by the decreased expression of genes associated with translation, transcription, secretion of immunogenic proteins, metabolism, and cell wall synthesis. Additionally, we identified distinguishing transcriptional effects specific to each antibiotic, indicating that different mechanisms of action induce distinct patterns in response to cellular injury. In addition to elucidating the *Mtb* physiologic changes associated with antibiotic stress, this study demonstrates the value of SEARCH-TB as a highly granular pharmacodynamic assay that reveals antibiotic effects that are not apparent based on culture alone.

## INTRODUCTION

Tuberculosis (TB) is the leading cause of death from infection globally, killing approximately 1.2 million people each year (1). Because standard antibiotic treatment regimens require 4 to 6 months to reliably cure drug-susceptible TB (2), there is an urgent need for new antibiotic combinations capable of curing all forms of TB more quickly (3). Mouse treatment models are a cornerstone of preclinical drug and regimen evaluation (4–6).

Colony-forming units (CFU) (7), a measure of bacterial burden in the lungs, are the pharmacodynamic (PD) marker historically used to evaluate drug efficacy in mice. However, change in CFU alone during treatment does not reliably distinguish between regimens that require different durations to achieve non-relapsing cure in the conventional mouse relapse model (7, 8). In addition to reducing bacterial burden, drug treatment also affects physiologic processes of *M. tuberculosis* (*Mtb*) (8, 9). It has been proposed that measurement of *Mtb* physiology during treatment could serve as an adjunctive PD approach, providing information distinct from bacterial burden (10). The effect of antibiotics on *Mtb* physiology has been studied extensively via transcriptional profiling *in vitro* (11–16) but not *in vivo*. This gap is important because exposure in axenic culture may not replicate the physicochemical conditions, host immunity, and dynamic pharmacokinetics encountered during *in vivo* exposure. To evaluate *Mtb* physiologic processes *in vivo*, we recently developed an RNA-seq platform, called SEARCH-TB (9). Via a combination of selective eukaryotic lysis to deplete host RNA, followed by targeted *Mtb* mRNA amplification, SEARCH-TB has unparalleled sensitivity for quantification of low-abundance *Mtb* transcripts, enabling transcriptional profiling of *Mtb* during the first month of drug treatment in mice (9).

With the long-term objective of developing SEARCH-TB as a preclinical PD marker for drug and regimen evaluation, we characterized *Mtb* that emerge during a 4-week treatment period with diverse antibiotics in mice. Our primary question was whether a single common shared *Mtb* persister transcriptome or drug-specific *Mtb* transcriptional patterns would emerge during 4 weeks of treatment with antibiotics that work by diverse mechanisms of action. After demonstrating drug-specific patterns, we then queried how transcriptional responses differed among individual drugs and between sterilizing and non-sterilizing drugs.

While all antibiotics included in conventional combination regimens are thought to contribute to cure to some degree, certain antibiotics play a more pronounced role in shortening the time required to cure TB (17). Historically, antibiotics, such as rifampin, pyrazinamide, and bedaquiline, which have potent treatment-shortening activity, have been described as “sterilizing,” while antibiotics, such as isoniazid, streptomycin, and ethambutol, which may have bactericidal activity but contribute only modestly to shortening the time needed to achieve a non-relapsing cure, have been described as “non-sterilizing” (18).

In this study, we compared the long-term effect of three canonical sterilizing antibiotics (rifampin, bedaquiline, and pyrazinamide) and three canonical non-sterilizing antibiotics (isoniazid, streptomycin, and ethambutol) over a 4-week treatment period in the BALB/c high-dose aerosol infection mouse model. We first identified *Mtb* transcriptional changes that were common to most of the antibiotics assessed, then compared the effect of sterilizing versus non-sterilizing antibiotics, and finally characterized transcriptional features unique to each antibiotic.

## MATERIALS AND METHODS

### Murine experiments and RNA extraction

Experiments used the BALB/c high-dose aerosol infection model, which is central to contemporary TB drug development (7). Female BALB/c mice, 6 to 8 weeks old, were exposed to aerosol (Glas-Col) with *Mtb* Erdman strain, resulting in 4.55 ± 0.03 (SEM) log_10_ CFU in the lungs on day 1. Mice euthanized after 11 and 19 days (when clinical deterioration met criteria for humane endpoints for euthanasia) served as pre-treatment and untreated control groups (*N* = 5 mice in each control group), respectively. Mice were treated via oral gavage 5 days a week starting on day 11. Mice were sacrificed on days 24–25 (*N* = 8 mice in each treatment group). Each mouse sample was profiled and analyzed separately without pooling of samples or data. We used established standard doses of all antibiotics (19) (Table S1), except for bedaquiline, which was administered at one-fifth of the standard dose because the full dose resulted in a *Mtb* burden that was too low for reliable profiling by SEARCH-TB. Lungs were flash-frozen before CFU enumeration and RNA extraction, as recently described (9).

### RNA sequencing and data preparation

Sequence analysis of samples was performed via SEARCH-TB following recently described methods (9). Briefly, RNA was reverse transcribed, and cDNA targets were then amplified using the SEARCH-TB panel. Libraries were sequenced on Illumina NovaSeq6000. We followed the bioinformatic analysis and quality control pipeline, as recently described (9).

### Statistical analysis

Following normalization with DESeq2’s variance stabilizing transformation (VST) (20), we performed a principal component analysis (PCA) on the 500 most variable genes. We estimated differential expression by fitting negative binomial generalized linear models to each gene using edgeR (21). Likelihood ratio tests were used to compare gene expression between groups.

To identify groups of genes with similar expression patterns across conditions, we performed a hierarchical clustering of the predicted expression values obtained from the edgeR models after filtering out invariant genes (i.e*.,* not differentially expressed between any two conditions). Then, using Euclidean distance with Ward’s method (22), we clustered the genes based on the predicted expression values for each condition. To further visualize the expression patterns for individual clusters, we used sample-specific, scaled VST-normalized expression values averaged across the genes in each cluster (Fig. S1). Using analysis of variance and post-hoc pairwise *t*-tests, we evaluated between-group differences for each cluster using these scaled expression values.

We performed a functional enrichment analysis using gene categories established by Cole et al. (23) and curated from the literature (Table S2) using the hypergeometric test in the hypeR package (24) to evaluate whether genes differentially expressed in pairwise comparisons between conditions were overrepresented in each gene set. Enrichment analysis was run twice using significantly upregulated and significantly downregulated genes separately. Gene categories with fewer than eight genes were excluded. All analyses were performed using R (v4.3.1), and comparisons were considered statistically significant when Benjamini–Hochberg adjusted *P*-values (25) were less than 0.05. Gene expression for individual gene categories was visualized using sample-specific scaled VST-normalized expression values averaged across the genes in the category (Fig. S2). Differential expression, functional enrichment, and visualizations can be evaluated interactively using an online analysis tool (https://microbialmetrics.org/analysis-tools/).

## RESULTS

### Effect of antibiotic treatments on CFU burden

**Fig 1:**
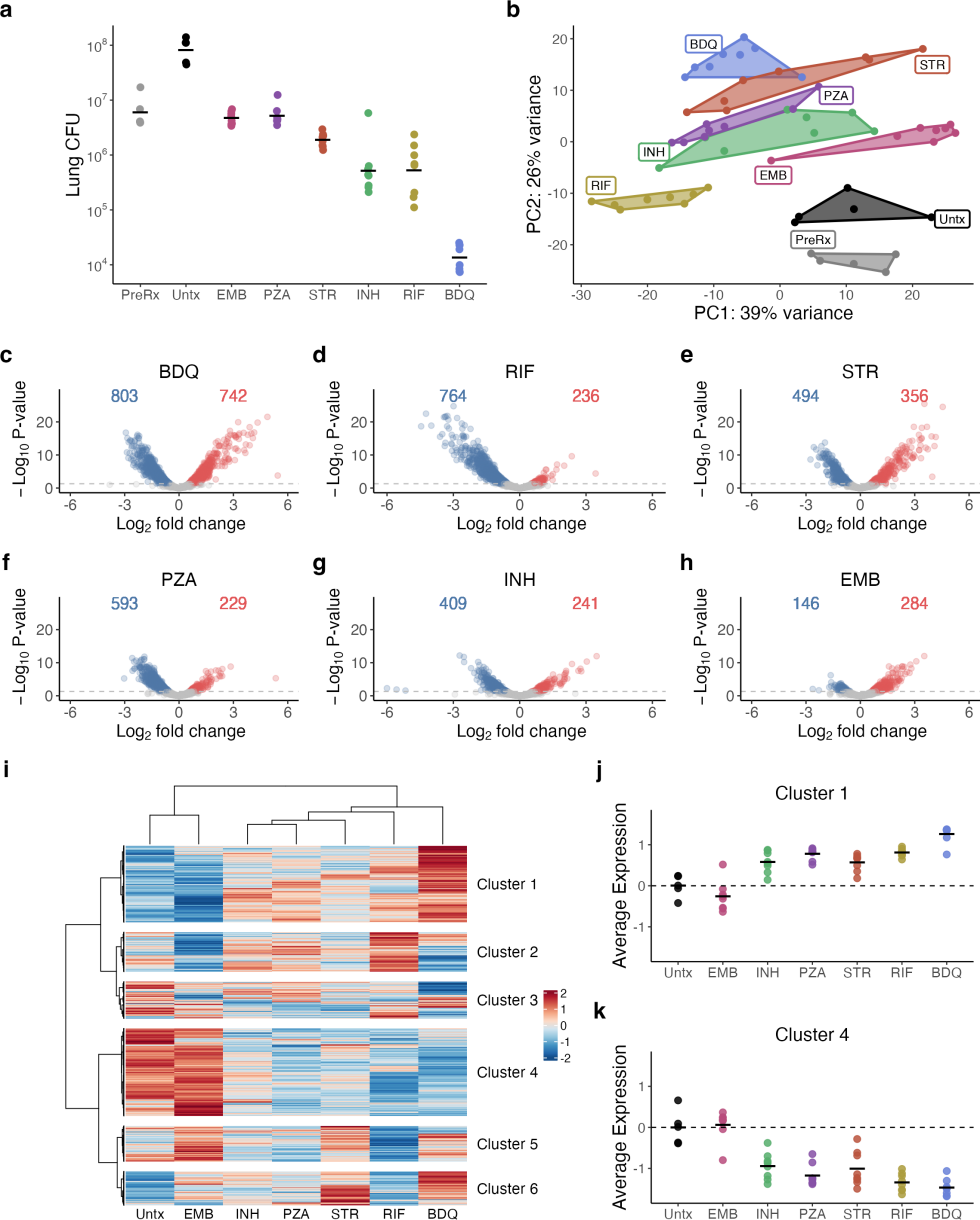
(**a**) *Mtb* CFU burden in the lungs of BALB/c mice after a 4-week treatment with individual antibiotics. Points indicate CFU values from individual mice. Horizontal bars indicate group means. PreRx and Untx indicate control mice sacrificed on the day treatment was initiated and 8 days thereafter, respectively. (**b**) Principal components analysis plot of VST-normalized gene expression data for the top 500 most variable genes. The first two principal components are shown on the x- and y-axes, and each point represents an individual sample. A convex hull highlights antibiotic treatments. (**c–h**) Volcano plots summarizing the differential expression between *Mtb* in untreated mice and *Mtb* in (**c**) BDQ, (**d**) RIF, (**e**) STR, (**f**) PZA, (**g**) INH, and (**h**) EMB. The number of genes significantly down- (blue) or up-regulated (red) for each antibiotic treatment relative to untreated (adj *P*-value < 0.05) are shown. (**i**) Heatmap of gene expression, including all genes significantly differentially expressed between at least two treatment conditions (*N* = 2,589). Expression values were obtained from edgeR models and then row-scaled, with red and blue indicating higher and lower expressions, respectively. Hierarchical clustering of genes identified six broad patterns. (**j, k**) Average of VST-normalized, scaled expression across treatments for clusters (**j**) 1 and (**k**) 4. Each point represents an individual mouse. Horizontal lines indicate average values. Values are centered around the average value for the untreated samples so that points above and below zero represent upregulation and downregulation relative to untreated, respectively. Abbreviations: untreated (Untx), ethambutol (EMB), isoniazid (INH), pyrazinamide (PZA), streptomycin (STR), rifampin (RIF), and bedaquiline (BDQ).

We first characterized the antibiotic effect based on changes in CFU, which estimates the number of bacilli capable of growth on solid agar (Fig. 1a). In pre-treatment control mice sacrificed on day 11 before the initiation of treatment, the average lung CFU burden was 6.78 log_10_. In untreated control mice, which were maintained without treatment until day 19 when clinical deterioration achieved humane endpoints for euthanasia, the average lung CFU burden was 7.91 log_10_. Pyrazinamide and ethambutol had a static effect, preventing an increase in CFU burden relative to the pre-treatment control but not reducing the CFU burden after 4 weeks of treatment. Streptomycin reduced lung CFU by 0.5 log_10_ relative to the pre-treatment control. Rifampin and isoniazid reduced CFU by 1.05 and 1.06 log_10_ relative to pre-treatment control, respectively. Bedaquiline had the greatest effect, reducing CFU by 2.64 log_10_.

### Clustering of antibiotic-induced transcriptional change

Principal component analysis of the SEARCH-TB results showed that samples from each antibiotic clustered distinctly from one another (Fig. 1b), demonstrating that antibiotics with unique mechanisms of action affect *Mtb* differently. The number of *Mtb* genes significantly altered relative to untreated control by antibiotic exposure ranged from 430 (ethambutol) to 1,545 (bedaquiline) (Fig. 1c through h), indicating that each antibiotic induced substantial changes in bacterial physiology. To visualize the differences between antibiotics, we performed unsupervised hierarchical clustering based on the expression of differentially expressed genes (Fig. 1i). Of the antibiotics evaluated, ethambutol was the most similar to the untreated control. Isoniazid, streptomycin, pyrazinamide, and rifampin clustered together and were distinct from the transcriptional changes caused by bedaquiline.

Unsupervised clustering of differentially expressed genes revealed that certain clusters of genes behaved concordantly among most antibiotics, while others behaved discordantly. For all antibiotics, except ethambutol, genes in Cluster 1 (*N* = 639) exhibited increased expression relative to untreated control (Fig. 1i). The magnitude of induction of Cluster 1 genes varied between antibiotics (Fig. 1j), with greater increase for bedaquiline than for any other antibiotic (*P-*value relative to the closest antibiotic = 0.0003). Conversely, for all antibiotics, except ethambutol, genes in Cluster 4 (*N* = 731) had decreased expression relative to the untreated control (Fig. 1i), with greater decreases for bedaquiline and rifampin than for isoniazid, streptomycin, and pyrazinamide (Fig. 1k). The remaining clusters (2, 3, 5, and 6) identified genes affected in distinct ways by antibiotics with different mechanisms of action (average expression plots in Fig. S2), consistent with the emergence of antibiotic-specific injury responses that are discussed further below. Functional enrichment for each cluster is summarized in File S1.

### Shared *Mtb* transcriptional responses to diverse antibiotic exposures

**Fig 2:**
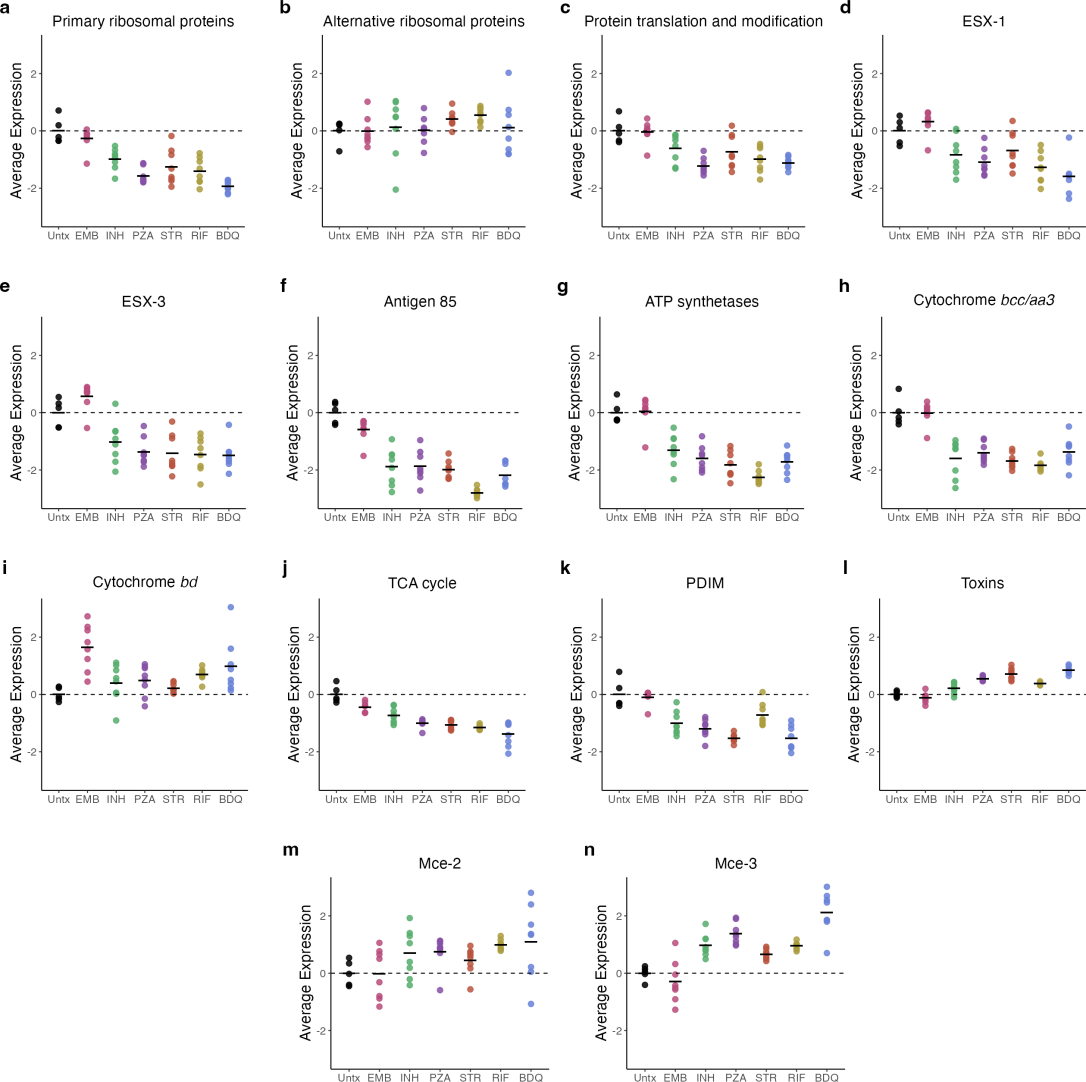
Average of VST-normalized, scaled gene expression across *Mtb* treatments in BALB/c mice for genes in key *Mtb* biological processes: (**a**) primary ribosomal proteins, (**b**) alternative ribosomal proteins, (**c**) protein translation and modification, (**d**) ESX-1, (**e**) ESX-3, (**f**) Antigen 85, (**g**) ATP synthesis, (**h**) cytochrome *bcc/aa3*, (**i**) cytochrome *bd*, (**j**) TCA cycle, (**k**) PDIM, (**l**) toxins, (**m**) MCE-1, and (**n**) MCE-3. Each point represents an individual mouse. Horizontal lines indicate average values. Values are centered around the average value for the untreated samples so that points above and below zero represent upregulation and downregulation relative to untreated, respectively. Abbreviations: untreated (Untx), ethambutol (EMB), isoniazid (INH), pyrazinamide (PZA), streptomycin (STR), rifampin (RIF), and bedaquiline (BDQ).

This section describes the transcriptional responses that were shared among most antibiotic exposures, relative to untreated control. As described above, ethambutol did not change CFU, clustered with the untreated control, and had the smallest number of differentially expressed genes relative to untreated control (Fig. 1h). To characterize effective antibiotic treatment, ethambutol was, therefore, excluded from our description of shared transcriptional responses below. For individual mice, we summarized the average normalized expression of genes in established biological categories (Table S2) (Fig. 2). Fig. S3 includes a corresponding heatmap that summarizes the average expression of individual genes for each category shown in Fig. 2. File S2 provides functional enrichment analysis *P*-values for all comparisons in this section. Comparisons described as significant had an adjusted *P*-value < 0.05.

#### Suppressed expression of genes associated with protein translation

Antibiotics concordantly decreased the expression of the primary ribosomal protein genes relative to the untreated control, consistent with slowing of protein synthesis (statistically significant in functional enrichment analysis for all antibiotics) (Fig. 2a). By contrast, the four “alternative” ribosomal protein genes involved in stress-induced ribosomal remodeling (26, 27) had sustained or increased expression (Fig. 2b) (gene set too small for statistical functional enrichment evaluation). Antibiotics decreased expression of the protein translation and modification category that includes genes responsible for translational initiation, promotion of tRNA binding, elongation, termination, and protein folding (Fig. 2c) (statistically significant in functional enrichment analysis for pyrazinamide, rifampin, and bedaquiline).

#### Decreased expression of immunogenic secretory proteins

Relative to untreated control, antibiotics decreased expression of the ESX-1 secretion system, including *esxA* and *esxB*, which encode the highly immunogenic early secretory antigenic 6 kDa (ESAT-6) and culture filtrate protein 10 (CFP-10), respectively (Fig. 2d) (statistically significant in functional enrichment analysis for all, except ethambutol and streptomycin). Antibiotics decreased expression of the ESX-3 system that secretes peptides that activate neutrophil and macrophages (Fig. 2e). Finally, antibiotics appeared to decrease expression of the three genes coding for the Antigen 85 complex (Fig. 2f), a secreted protein essential for survival within macrophages, which also helps to maintain the *Mtb* cell wall integrity by catalyzing the transfer of mycolic acids to cell wall (gene set too small for statistical functional enrichment analysis) (28).

#### Metabolic slowing and adaptation

Relative to the untreated control, antibiotics significantly suppressed expression of genes coding for ATP synthetases (Fig. 2g). Oxidative phosphorylation appeared to transition from the primary cytochrome *bcc/aa3* supercomplex (downregulated) to the less-efficient cytochrome *bd* oxidase (upregulated), which has been implicated in persistence under environmental and antibiotic stress (29) (Fig. 2h and i) (gene sets too small for statistical functional enrichment evaluation). Antibiotics were associated with decreased expression of TCA cycle genes (Fig. 2j) (all, except ethambutol and rifampin, were statistically significant in functional enrichment analysis). Respiratory slowing was not accompanied by the expected increased expression of glyoxylate bypass genes, an alternative pathway previously implicated in antibiotic tolerance (30). Genes associated with carbon storage, such as triacylglycerol, were also not upregulated. Specifically, *tgs1*, a gene in the DosR regulon, which codes for triacylglycerol synthase previously associated with lipid accumulation during treatment (31), had significantly decreased expression after exposure to all drugs, except ethambutol and isoniazid (see the online analysis tool).

#### Decreased synthesis of mycolic acids and PDIM

Antibiotics significantly reduced the expression of Rv2524c (*fas*), the gene coding for fatty acid synthetase I, indicating a slowdown in the first step of mycolic acid synthesis (see the online analysis tool). All antibiotics, except ethambutol, appeared to decrease expression of phthiocerol dimycocerosate (PDIM), suggesting potential decreased virulence of the antibiotic-stressed phenotypes (32) (Fig. 2k) (statistically significant in functional enrichment analysis for all antibiotics, except ethambutol and rifampin).

#### Regulation of growth: sigma factors

Consistent with transition to a quiescent phenotype, antibiotics resulted in significantly lower expression of *sigA*, which codes for the primary ‘housekeeping’ sigma factor necessary for growth, relative to untreated control (see the online analysis tool). Other sigma factors were affected differently by individual antibiotics and are discussed in Section 5 below.

#### Modulation of stress responses

Antibiotics induced expression of genes for toxins that act post-transcriptionally to reprogram *Mtb* in response to stress (Fig. 2l) (statistically significant in functional enrichment analysis for streptomycin, pyrazinamide, and bedaquiline). However, as described below, the pattern of which toxin genes had increased expression differed depending on antibiotic exposure. Consistent with the change previously observed with the standard four-drug regimen (9), mammalian cell entry (*mce*) operons initially identified as *Mtb* virulence adaptations and more recently implicated in stress adaptation (33) appeared to have increased expression of Mce-2 and Mce-3 operons with all drugs, except ethambutol (Fig. 2m and n) (gene sets too small for statistical functional enrichment evaluation).

### Transcriptional response to sterilizing versus non-sterilizing antibiotics

This section summarizes pairwise comparisons between drugs rather than comparison between drugs and untreated control. File S3 provides functional enrichment analysis *P-*values for all comparisons in this section. Comparisons described as significant had an adjusted *P*-value < 0.05

Comparison of canonical sterilizing antibiotics (rifampin, pyrazinamide, bedaquiline) with non-sterilizing antibiotics (isoniazid, streptomycin, ethambutol) suggests that sterilizing drugs generate a more quiescent *Mtb* phenotype defined by decreased expression of growth-associated genes coding for translation, transcription, secretion of immunogenic proteins, metabolism, and cell wall synthesis. Specifically, expression of genes coding for primary ribosomal proteins, a basic metric of bacterial activity, was suppressed to a significantly greater degree by bedaquiline than by the non-sterilizing antibiotics isoniazid, streptomycin, and ethambutol (Fig. 2a). Rifampin and pyrazinamide suppressed primary ribosomal protein gene expression significantly more than two (isoniazid, ethambutol) of three non-sterilizing antibiotics. As discussed above, expression of the protein translation and modification gene category was decreased significantly for the sterilizing antibiotics but not for the non-sterilizing antibiotics. Expression of the gene for the RNA polymerase subunit A (*rpoA*) was significantly decreased by all sterilizing antibiotics but not by any non-sterilizing antibiotics. Similarly, RNA polymerase subunit Z (*rpoZ*) was significantly decreased by all sterilizing antibiotics and only one (isoniazid) of the non-sterilizing antibiotics. All three sterilizing antibiotics had significantly decreased expression of *esxA*, the gene coding for ESAT-6 relative to isoniazid and ethambutol. Expression of the gene coding for isocitrate lyase (*icl1*), the first step of the glyoxylate bypass, was decreased significantly by all three sterilizing antibiotics but by none of the non-sterilizing antibiotics.

**Fig 3:**
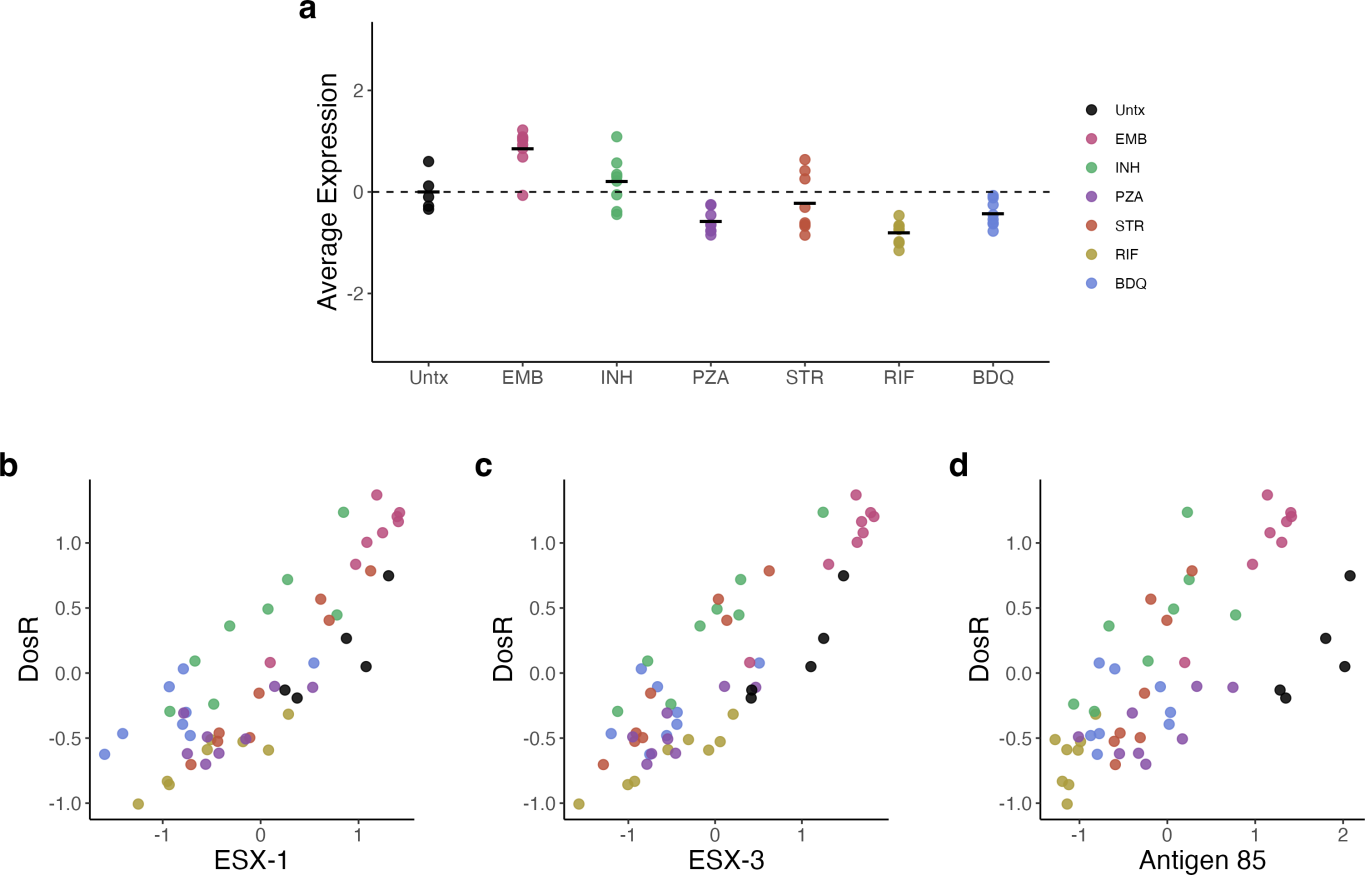
(**a**) Average of VST-normalized, scaled expression across antibiotic treatments for genes in the DosR regulon. (**b-d**) Correlation between the scaled average expression for categories associated with immune activation and DosR: ESX-1, ESX-3, and Antigen 85. Each point represents an individual mouse, and points are colored by treatment group. Abbreviations: untreated (Untx), ethambutol (EMB), isoniazid (INH), pyrazinamide (PZA), streptomycin (STR), rifampin (RIF), and bedaquiline (BDQ).

Expression of DosR regulon genes, which respond to hypoxia, carbon monoxide, and nitric oxide encountered within host immune effector cells, was significantly reduced by all sterilizing drugs but not by the non-sterilizing drugs (Fig. 3a). Because bacterial DosR expression has previously been linked to the intensity of immune activation (9, 34), we plotted the average normalized expression values for the ESX-1, ESX-3, and Antigen 85 genes against average normalized expression of DosR regulon genes (Fig. 3b through d). Expressions of ESX-1, ESX-3, and Antigen 85 were correlated with expression of the DosR regulon (R^2^ = 0.7, R^2^ = 0.745, and R^2^ = 0.5, respectively), suggesting a link between bacterial phenotype and immune activation.

### Distinguishing effects of individual antibiotics

**Fig 4:**
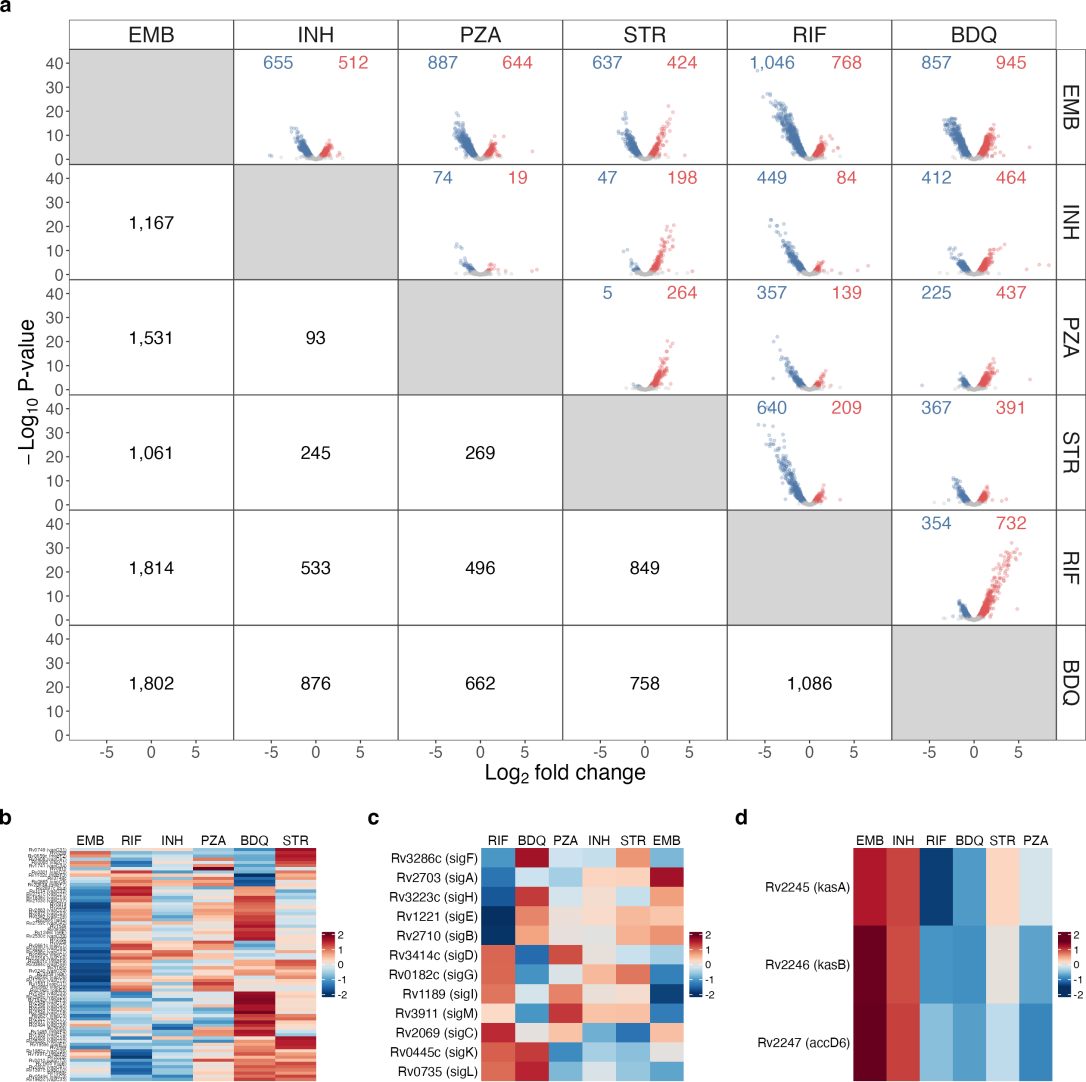
(**a**) Differential expression in pairwise comparison between individual antibiotics. Volcano plots show fold change and significance between the antibiotics labeled in the row and column. The number of genes significantly down- (blue) or upregulated (red) with adjusted *P*-value < 0.05 in the row versus the column is shown below the diagonal. (**b–d**) Heatmaps showing the VST-normalized, scaled average expression across antibiotic conditions for (**b**) toxins, (**c**) sigma factors, and (**d**) the *kas* operon. Abbreviations: ethambutol (EMB), isoniazid (INH), pyrazinamide (PZA), streptomycin (STR), rifampin (RIF), and bedaquiline (BDQ).

Finally, we considered differences in transcriptional changes induced by each individual antibiotic exposure. Despite the existence of shared transcriptional changes discussed above, direct pairwise comparison between antibiotic exposures revealed that each antibiotic resulted in a distinct *Mtb* transcriptional response (Fig. 4a). File S3 provides functional enrichment analysis *P-*values for all comparisons in this section. Comparisons described as significant had an adjusted *P*-value < 0.05. Key observations from these tables are highlighted below.

#### Bedaquiline

Although evaluated at one-fifth the standard dose, bedaquiline induced the greatest transcriptional change of any antibiotic, significantly altering expression of 1,545 genes relative to untreated control (Fig. 1c). The bedaquiline-treated phenotype was distinct, with at least 662 genes differentially expressed relative to any other antibiotic (bottom row of Fig. 4a). Bedaquiline-mediated inhibition of ATP synthetase led to a profoundly quiescent *Mtb* population, consistent with an energy-restricted phenotype. Specifically, relative to all antibiotics other than pyrazinamide, bedaquiline significantly decreased the expression of genes coding for primary ribosomal proteins and genes associated with the synthesis and modification of macromolecules. Bedaquiline suppressed the ESX1 locus to a significantly greater degree than isoniazid, streptomycin, or ethambutol. Additionally, bedaquiline induced greater expression of certain stress responses. Specifically, bedaquiline induced significantly greater expression of genes for stress-induced toxin/antitoxin modules, relative to any antibiotic other than streptomycin (Fig. 4b). Relative to any other antibiotic, bedaquiline also induced a greater expression of sigma factor F, which directs growth arrest in response to diverse stresses (Fig. 4c) (35).

#### Rifampin

Evaluated at the existing standard dose, rifampin had the second-strongest effect on the *Mtb* transcriptome, significantly altering the expression of 1,000 genes relative to untreated control (Fig. 1d). The rifampin-treated phenotype was distinct, with at least 496 genes differentially expressed relative to any other antibiotic (second from bottom row of Fig. 4a). Rifampin resulted in significantly higher expression of genes involved in the cell wall than all antibiotics, except ethambutol, and significantly higher expression of PDIM than all antibiotics, except ethambutol and isoniazid. Rifampin had significantly lower expression of the primary housekeeping sigma factor A than any antibiotic other than pyrazinamide, consistent with the regulation of a quiescent phenotype (Fig. 4c and online analysis tool). Rifampin was distinct from all other antibiotics in having significantly increased expression of *sigE*, which codes for sigma factor E that mediates slower growth under stress conditions (36). All other antibiotics had significantly decreased expression of *sigE*. Rifampin resulted in significantly lower expression of genes coding for chaperones and heat shock proteins and the enduring hypoxic response (37) than any other antibiotic. Rifampin-treated *Mtb* had significantly lower expression of the DosR regulon than *Mtb* treated with any antibiotic, except bedaquiline.

#### Pyrazinamide

Pyrazinamide treatment resulted in broad changes in the *Mtb* transcriptome, significantly altering the expression of 822 genes relative to untreated control (Fig. 1f). Relative to rifampin, pyrazinamide had significantly higher expression of genes coding for the DosR regulon and the Antigen 85 complex, as well as genes involved in beta-oxidation, electron transport, and toxin–antitoxin modules. Pyrazinamide clustered with isoniazid based on global similarity (Fig. 1b and i), and relatively few genes were differentially expressed between pyrazinamide and isoniazid (96 significant genes, Fig. 4a), yet the pyrazinamide phenotype appeared more quiescent than the isoniazid phenotype, with significantly lower expression of genes involved in protein translation and modification, ribosomal protein synthesis, and synthesis and modification of macromolecules.

#### Isoniazid

Isoniazid significantly altered the expression of 650 genes relative to untreated control (Fig. 1g). Isoniazid exhibited higher expression of the mycolic acid synthesis genes of the *kas* operon than any antibiotic other than ethambutol, suggesting continuing *Mtb* compensation to the isoniazid mechanism of action (Fig. 4d and online analysis tool). Isoniazid also had significantly higher expression of DosR regulon genes compared to all antibiotics, except ethambutol, suggesting adaptation to continued immune-mediated nitric oxide or hypoxic stress.

#### Streptomycin

Streptomycin significantly altered the expression of 850 genes relative to untreated control (Fig. 1e). The streptomycin phenotype was distinct, with at least 245 genes differentially expressed relative to any other antibiotic (Fig. 4a). Protein synthesis inhibition by streptomycin resulted in significantly higher expression of toxin–antitoxin pairs and of the enduring hypoxic response compared to any antibiotic other than bedaquiline. Streptomycin also resulted in significantly higher expression of chaperones and heat shock genes compared to any antibiotic other than ethambutol and significantly higher expression of genes associated with the response to oxidative stress than any antibiotic other than ethambutol or bedaquiline.

#### Ethambutol

Ethambutol induced the least transcriptional change among the antibiotics assessed, with 430 genes significantly altered relative to untreated control (Fig. 1h). The ethambutol transcriptome clustered with the untreated control (Fig. 1i) and was distinct from other antibiotics in most of the discrete processes shown in Fig. 2.

## DISCUSSION

We found that treating mice for 4 weeks with one of six antibiotics representing distinct mechanisms of action led to emergence of both shared and antibiotic-specific *Mtb* transcriptional responses. Antibiotics differed both in the magnitude of transcriptional change they induced in *Mtb* and the specific sets of genes up- or downregulated. Broadly, rifampin, pyrazinamide, and bedaquiline, the antibiotics with enhanced treatment-shortening activity (historically described as sterilizing), led to a more quiescent bacterial phenotype than did antibiotics with lesser treatment-shortening activity (historically described as non-sterilizing).

*Mtb* subpopulations that are genotypically drug-susceptible yet survive extended drug exposure *in vivo* are viewed as a central obstacle to shortening the time required to cure TB (38, 39). Our results suggest that different individual drugs result in distinct *in vivo* “persister” *Mtb* phenotypes. Antibiotics with different mechanisms of action impart distinct injuries that condition the physiologic processes of *Mtb* in distinct ways. While some broad transcriptional responses are shared among antibiotics (e.g*.,* downregulation of genes associated with synthesis of macromolecules and metabolism and upregulation of certain stress responses), each antibiotic also had unique effects on the *Mtb* transcriptome.

Of particular interest are sterilizing antibiotics known to play an outsized contribution to the ability of combination regimens to shorten the time required to cure TB. In this study, we selected three antibiotics with enhanced treatment-shortening activity—rifampin, pyrazinamide, and bedaquiline—that are central to contemporary regimen development and are included in recent and ongoing human trials. The SEARCH-TB analysis revealed that rifampin, pyrazinamide, and bedaquiline resulted in a more quiescent phenotype than did isoniazid, streptomycin, and ethambutol. This finding aligns with our previous observations using the RS ratio assay in the same mouse sample set, which showed that rifampin, pyrazinamide, and bedaquiline decreased ribosomal RNA synthesis to a greater degree than antibiotics with lesser treatment-shortening activity (8). Combined with the RS ratio results, the SEARCH-TB data suggest that a common effect of antibiotics with potent treatment-shortening activity is the induction of a more quiescent *Mtb* phenotype. Our findings suggest but cannot definitively resolve two potential interpretations for the observed association between treatment-shortening activity and a more quiescent phenotype. First, a more quiescent phenotype may represent a functional physiologic adaptation that enables *Mtb* to survive exposure to rifampin, pyrazinamide, or bedaquiline but is less crucial for surviving isoniazid, streptomycin, and ethambutol. Alternatively, the more quiescent phenotype could be a “vital sign” of bacterial injury, signaling more severe stress and resultant bacterial dysfunction. A *Mtb* population experiencing energy starvation (bedaquiline) or transcriptional inhibition (rifampin) may be functionally incapacitated or in a pre-terminal state.

Certain drugs had a larger magnitude of effect on the *Mtb* transcriptome than others. It seems notable that ethambutol, a drug that contributes negligibly to the activity of HRZE (18), had the smallest number of differentially expressed genes relative to control. In unsupervised clustering, the transcriptome of ethambutol-treated *Mtb* was similar to pre-treatment control, suggesting that ethambutol had a smaller effect on *Mtb* physiology than any other drug. Conversely, even when administered here at 1/5 the standard dose, bedaquiline had the largest number of differentially expressed genes and was an outlier in unsupervised clustering with more extreme expression changes. This seems concordant with previous observations that bedaquiline has greater sterilizing activity than first-line drugs in murine models (40) and has transformed treatment of human multidrug- and extensively drug-resistant TB (41, 42).

We note that drugs with distinct mechanisms of action (e.g*.,* isoniazid and pyrazinamide) were proximal in PCA and hierarchical clustering. Important to interpretation of our SEARCH-TB results is the distinction between the direct mechanism by which an antibiotic engages its target protein and the resulting indirect cascade of physiologic injury and adaptation. For example, the direct engagement of bedaquiline with the *Mtb* ATP synthetase enzyme is not immediately lethal (43, 44). Instead, this engagement initiates a cascade of metabolic changes previously described as “respiratory dysfunction” (43). *In vitro,* this cascade progresses over days, culminating in loss of homeostasis, collapse of the transmembrane pH gradient, and lysis as a final terminal event (43). Since our study collected samples after 4 weeks of treatment rather than at the initiation of treatment, our SEARCH-TB results likely quantify the pattern of drug-induced injury and adaptation (i.e*.,* an indirect consequence of drug exposure) rather than the immediate effect of drug-target engagement. We speculate that this may explain why drug exposures cluster in ways that are not immediately explicable based on drug mechanism alone.

Rifampin, pyrazinamide, and bedaquiline significantly decreased the expression of the DosR regulon relative to the untreated control mice. This result is consistent with previous observations that HRZE decreased DosR expression in mouse lung (9) and human sputum (34). However, it conflicts with previous reports that bedaquiline and HRZE increased DosR expression *in vitro* (9, 44). Since the mechanisms of action of antibiotics tested here do not directly target the known drivers of DosR expression (hypoxia, nitric oxide, and carbon monoxide), decreased DosR regulon can be considered an indirect effect of treatment *in vivo*. The discrepancy between *in vitro* and *in vivo* results suggests that expression of DosR during treatment might be modulated by the presence of immunity. A link between macrophage activation state, nitric oxide exposure, and DosR expression is well established (45, 46). Activation of macrophages was shown to induce DosR regulon expression in the presence of functional host nitric oxide synthase 2 (NOS2) but not in NOS2-deficient macrophages (47, 48). If drug treatment decreases macrophage activation state in mice, as is suggested by human blood transcriptome studies (49, 50), the resultant decreased nitric oxide exposure might explain decreased *Mtb* expression of DosR regulon genes. Future studies will evaluate effect of treatment on macrophage activation and *Mtb* DosR expression.

This work highlights the power of SEARCH-TB as a pharmacodynamic marker. It has long been recognized that a major impediment for the study of TB drugs in mice is the limitations of the CFU readout (7, 51). Specifically, because bactericidal activity (reduction in CFU) does not reliably indicate sterilizing activity in the conventional BALB/c high-dose aerosol model, large-scale relapse studies are required to estimate sterilizing activity (7, 51). The failure of CFU to predict sterilizing activity in the BALB/c mouse is highlighted by our observation that week four CFU did not distinguish between the effects of pyrazinamide (potent sterilizing activity [18]) and ethambutol (minimal sterilizing activity [18]). By contrast, SEARCH-TB showed that pyrazinamide and ethambutol treatment resulted in 1,531 genes or 43% of the transcriptome differentially expressed, indicating profoundly different effects on *Mtb* physiology. Similarly, while CFU failed to distinguish between the effects of rifampin (potent sterilizing activity [18]) and isoniazid (minimal sterilizing activity [18]), SEARCH-TB showed that rifampin and isoniazid affect *Mtb* physiology differently (533 differentially expressed genes). Our results demonstrate that SEARCH-TB can elucidate drug effects that are not discernible based on CFU alone. This work is an incremental step toward our long-term goal of developing SEARCH-TB as a highly granular PD marker in preclinical drug and regimen assessment. Future projects will evaluate the ability of SEARCH-TB to probe TB drug interactions *in vivo* and predict regiment treatment-shortening potency.

This report has several limitations. First, this report characterized drug-induced phenotypic change in the lungs of BALB/c mice, which develop loose macrophage aggregates containing intracellular *Mtb*. Other TB mouse models, such as the C3HeB/FeJ mouse, develop necrotic granulomas in which *Mtb* is extracellular and has distinct phenotypic adaptations to local conditions (52). A high-priority next step is interrogating *Mtb* in diverse models to elucidate the full spectrum of bacterial phenotypes and antibiotic responses. Second, we used the high-dose aerosol infection model because it is a mainstay of contemporary preclinical drug and regimen evaluation (7). High-dose aerosol infection is lethal if mice are not “rescued” by initiation of antibiotic treatment (53). In this experiment, untreated mice experienced clinical deterioration necessitating humane euthanasia 19 days after aerosol infection. The untreated control, therefore, could not be temporally matched with the antibiotic-treated mice. Third, all antibiotics were evaluated at a human-to-mouse adjusted equivalent dose, except bedaquiline, which reduced *Mtb* burden below the limits of SEARCH-TB reliability with human equivalent dosing with 4 weeks of treatment. It is likely that higher or lower drug doses might induce different transcriptional responses, which remains to be tested. Finally, SEARCH-TB quantifies expression across the entire lung, inherently representing a population average that does not reveal heterogeneity that likely exists within the population.

Using a novel pathogen-targeted RNA-seq method, we evaluated *Mtb* after 4 weeks of treatment with individual antibiotics *in vivo*, demonstrating that antibiotics with different mechanisms of action lead to distinct *Mtb* phenotypes. Sterilizing antibiotics generated a more quiescent *Mtb* phenotype than non-sterilizing drugs. This report demonstrates the capability of SEARCH-TB to reveal differences in antibiotic effects that are not discernible via conventional microbiologic tools, potentially enabling a new era of pharmacodynamic monitoring in which candidate TB treatments are evaluated *in vivo* based on highly granular assessment of bacterial physiologic processes.

## Data Availability

All raw sequencing data have been deposited in the Sequence Read Archive (SRA) under BioProject accession PRJNA1188116. Individual samples have BioSample accession numbers SAMN44825236 through SAMN44825293.

## SUPPLEMENTAL MATERIAL

File S1 aac.01310-24-s0001.xlsx Categorical enrichment for clusters of genes found using unsupervised clustering.

File S2 aac.01310-24-s0002.xlsx Categorical enrichment of each antibiotic relative to control.

File S3 aac.01310-24-s0003.xlsx Categorical enrichment of each antibiotic relative to one another.

Supplemental material aac.01310-24-s0004.docx Supplemental methods, Tables S1 and S2, and Figures S1 to S3.

ASM does not own the copyrights to Supplemental Material that may be linked to, or accessed through, an article. The authors have granted ASM a non-exclusive, world-wide license to publish the Supplemental Material files. Please contact the corresponding author directly for reuse.
